# Measures of the psychophysiological response to recurrent anticipatory stress - the influence of neuroticism on heart rhythm and skin resistance

**DOI:** 10.1038/s41598-025-28090-7

**Published:** 2025-11-21

**Authors:** Florestan Wagenblast, Robert Seibt, Monika A. Rieger, Benjamin Steinhilber

**Affiliations:** Institute of Occupational and Social Medicine and Health Services Research, University Hospital Tübingen (UKT), Wilhelmstraße 27, 72074 Tübingen, Germany

**Keywords:** Risk assessment, Psychophysiology, Physiological adaptation, Neuroticism, Heart rate, Galvanic skin response, Health occupations, Risk factors

## Abstract

In the risk assessment of work-related mental stress, the use of objective psychophysiological methods could improve the identification of mental stressors. However, mental stressors often reoccur during work, and adaptation processes and individual factors may alter psychophysiological responses over time. In this exploratory study, measures of heart rhythm and skin resistance during recurrent anticipatory stress were examined in two groups with lower and higher neuroticism to provide information on which method is most appropriate for stress detection. In the with-in subject design study participants were required to first anticipate a neutral audio signal during a control condition and then a potential painful electric shock in two subsequent experimental conditions. Heart rate (HR), heart rate variability (HRV), and skin resistance (SR) parameters were extracted during anticipation. Groups with lower and higher neuroticism were formed based on the Big Five Inventory. While SR parameters and the low-frequency HRV responded similarly under both electric shock conditions, the root mean square of successive time differences between heartbeats, and the high-frequency HRV did not respond. HR increased in the 1st electric shock condition in the higher neuroticism group only. Parameters that responded under both electric shock conditions may contribute to more accurately mapping of recurrent anticipatory stressors.

**Supplementary Information:**

The online version contains supplementary material available at 10.1038/s41598-025-28090-7.

## Introduction

According to DIN EN ISO 10075 mental stress at work acts upon an individual when an event or task (stressor) is upcoming or ongoing and results in mental strain associated with either positive and/or negative effects. If mental stress is perceived as a threat to the individual’s integrity (physical and mental), it is usually accompanied by a negative affect^1^. The individually resulting mental strain can affect individuals’ safety at work in the short term^2^ and individuals’ ability to work in the longer term. The long-term effects can be explained, for example, through an increased risk of several health problems such as cardiovascular diseases or musculoskeletal, mental and behavioral disorders^3^. At the societal level the total cost of work-related depression alone has been estimated at up to €617 billion per year in 2013 in the European Union^4^.

In this respect, the German Occupational Health and Safety Act stipulates that employers are obliged to carry out a risk assessment of mental stress at the workplace. This incorporates the identification of stressors together with an assessment of the potential health risk due to mental strain. Especially for the latter purpose, mainly subjective methods (e.g. surveys) are used, which are in contrast to the risk assessment of physical stress, where the concept of an objective sensor-based risk assessment is gaining importance^5^. Since subjective methods present methodical limitations and psychological issues like scaling effects, recall bias, social desirability, etc.^6^, the use of additional psychophysiological methods that might contribute to an objective assessment of mental strain offer the chance to increase the construct validity of mental stress at work. Further, stressors may be mapped and tracked more accurately through real-time recordings^7–9^. Thus, for example, field studies could be used more effectively to develop preventive strategies for work design.

A frequently used method is to derive the heart rate (HR) and heart rate variability (HRV) from an electrocardiogram or recordings of the pulse wave, since it is relatively easy to apply in many contexts and discriminates work conditions with lower and higher mental demands^10–12^. Further, HRV is considered a biomarker of mental strain at the workplace^13,14^. This is because theories suggest that several HRV parameters are correlated with the vagal tone^15^. Reduced vagal tone may reflect a decrease in parasympathetic activity, which is associated with limited (autonomous) adaptive capacity to present stressors^16,17^. In order to increase the field of application, some parameters derived from the time and frequency domains of successive beat-to-beat RR intervals have recently been positively checked for their validity in detecting a response to mental stress using ultra-short-term recordings (< 5 min). These include for a minimum recording time of two minutes the mean HR (mHR), the root mean square of successive differences of the time period between normal heartbeats (RMSSD), and the low and high frequency domains (LF and HF) of the HRV^18–23^.

A further method is to determine the electrodermal activity (EDA) of the skin through exosomatic measurements of the skin conductance (SC) or the skin resistance (SR)^24^. Measures of EDA demonstrated to be a valid index of immediate sympathetic nervous system reactivity particularly in response to affective stimuli^25^ that can elicit emotions based on appraisal components and processes^26^. For example, it was shown that EDA responses can distinguish low-risk and high-risk situations in construction workers^27^. However, in comparison to measurements of the heart rhythm, EDA is less often assessed in field studies due to several limitations regarding possible influences by physical activity, time of day, or climate, among others^11,28,29^. In the future, new technical and analytical approaches may overcome such limitations^30,31^. Therefore, well-known manufacturers of wearable tracking devices rely on HRV, HR and EDA for stress detection^32^.

EDA might be useful as a complementary measurement because it directly depends on sympathetic activation of the eccrine sweat glands, mediated by the sudomotor nerve^33^, while HR or HRV reflect interdependent regulatory circuits, such as the autonomic balance between the sympathetic and parasympathetic activity, temperature regulation, or blood pressure control etc.^34^. This makes it difficult in field measurements of HR and HRV to isolate the mentally-induced physiological response in the presence of a physical stressor, such as heat^35^. Further, cerebral activation patterns of EDA were found to be distinct from those associated with responses of the HR or HRV refuting the assumption that the autonomic nervous system (ANS) respond to stressors as an unitary system^36^.

While many studies investigated physiological responses during or after (work-related) stressors, less attention has been paid to the period when a stressor is anticipated. Although even the appraisal of a stressor, including the expected potential physiological, affective or cognitive consequences, can trigger stress responses via feedforward loops^37,38^. This involves higher-order processing to estimate the individual significance of the anticipated stress^39^. At work, for example, this could be an upcoming meeting, a performance review by a supervisor, or safety hazards associated with physical pain or injury^40^. The appraisal depends heavily on the level of information about and experience with a stressor and therefore may change responsiveness to the same reoccurring stressors. Adaptions over time in the responsiveness, for example, can be the result of habituation and sensitization effects^41^, which can vary among stress measurements related to the ANS and may depend on personal factors as well^42^. This is particularly important for a mental stress assessment at work, where stressors can recur over time. Further, it was shown that anticipation of a stressor can have an immediate negative impact on cognitive or executive functions^43,44^ that might impair safety at work.

In general, previous research indicates that the acute reactivity to stress also depends on individual factors like the personality. A personality trait that has been often associated with stress reactivity is neuroticism. Costa and McCrae^45^ described neuroticism as a tendency to experience negative, distressing emotions and to possess associated behavioral and cognitive traits like fearfulness among others. With this in mind, neuroticism may influence the acute stress response in two ways. Firstly, given that stress responses to anticipatory stress can highly depend on appraisal processes, it may be positively correlated with the resulting mental strain. In individuals presenting high values of neuroticism negative emotions may be amplified because stress is perceived as incontrollable due to the feeling of an inability to cope^46,47^. Secondly, research showed that higher neuroticism is linked to a blunted stress reactivity regarding cardiovascular and cortisol responses, for example during the Trier Social Stress Test, which may be the result of functional dysregulations in the limbic system^48^.

The present exploratory study was primarily conducted to examine which psychophysiological measures capture acute physiological reactivity related to mental strain to recurrent anticipatory stress. Secondary, psychophysiological measures were examined in two groups with lower and higher neuroticism to gain insights into potential moderating effects of personality traits. Answering these questions could provide valuable information which objective parameter may be most useful to supplement risk assessment approaches of mental stress. Overall, it was intended to identify and discuss methodological challenges when psychophysiological measurements are used for the risk assessment of mental stress in the workplace and to derive hypotheses for future studies.

## Materials and methods

In this exploratory study 53 women and men were included and divided in two groups of lower and higher neuroticism. Using a within subject design, physiological responses during a control condition and recurrent acute anticipatory stress induced by the possibility of a painful electric shock were compared depending on the personality trait neuroticism. This approach was contemplated to investigate methodological potentials of psychophysiological methods for a risk assessment of work-related mental stress.

### Participants

It was intended to recruit 60 participants from three age groups (18–30, 31–55, 56–67 years), allowing for a balanced gender ratio (30 women, 30 men). Due to time and resource constraints, 53 healthy persons (30 women, 23 men) finally participated which were recruited mainly via e-mail distributors of the University Hospital and the University of Tübingen (Germany). Exclusion criteria were assessed anamnestically by experimenters, and included being under 18 or over 67 years of age, a body mass index under 18 or above 30, heart problems, neurological, psychological, or metabolic diseases, and the use of medicines including beta-blockers, analgesics, antipsychotics, antidepressants, anticonvulsants, or anxiolytics. All participants received financial compensation and gave their written, informed consent to participate in the study. The study was approved by the ethical committee of the medical Faculty of the University of Tübingen (561/2016BO1) and carried out according to the principles of the Declaration of Helsinki (version 2013, Fortaleza).

### Design and procedure

The participants were advised to avoid sports activities involving the upper body and alcohol consumption one day prior to the measurement. On the day of the measurement, sports and heavy physical work were not allowed, as well as caffeine, cocoa or any other drug consumption. From two hours before the start of the measurement eating or drinking (except water) were also not allowed. Measurements were completed for each participant at one appointment and lasted approximately two hours. They filled out the Big Five Inventory-SEOP and were prepared for measurements. This included, in addition to measurements of the heart rhythm and SR, surface electromyography at the trapezius and frontalis muscle, as well subjective ratings of the perceived mental stress. The results of the two latter measurements have been previously published^49^.

During the measurements light and temperature (22–25 °C) were kept largely constant, ambient noise was reduced to a minimum by closing the windows and the soundproof door of the room, and participants sat comfortably on an office chair in front of a desk with a computer screen. The setup was individually adjusted to the body height to ensure a knee and hip flexion angle of about 90° and that the upper edge of the screen was at eye level. During the recordings, the participants followed these instructions: to keep eyes on the screen, to breathe easily, to avoid movements. Visual contact between the examiner was prevented by a room divider, and experimental guidance was provided on-screen using a standardized slide show.

The study followed a within subject design and comprised three conditions in the following order: a control condition with the expectation of an audio signal (NoShock), a first experimental condition with the expectation of an electric shock (Shock1) to find out which measure is able to capture a response to the anticipatory stressor, and a second experimental condition with the expectation of the same electric shock (Shock2) to find out if a response can be captured for a second time and may be influenced by adaption effects^50^. Each condition was completed once by each participant and included an expectation phase, the application of the stimulus, and a subsequent 4-min recovery phase. Right before each expectation phase, the participants verbally rated their perceived mental stress related to the expectation of the stimulus on a numeric scale. In NoShock a pleasant audio signal was expected, which was introduced to the participants in advance. The exact timing of the stimuli, including the audio signal, and the intensity of the electric shocks were unknown to the participants. During a period of up to 20 min between the conditions NoShock and Shock1, the intensity of the electric shock was determined individually. The time between the end of Shock1 recovery phase and the start of Shock2 expectation phase was about 1 min.

**Fig. 1:**
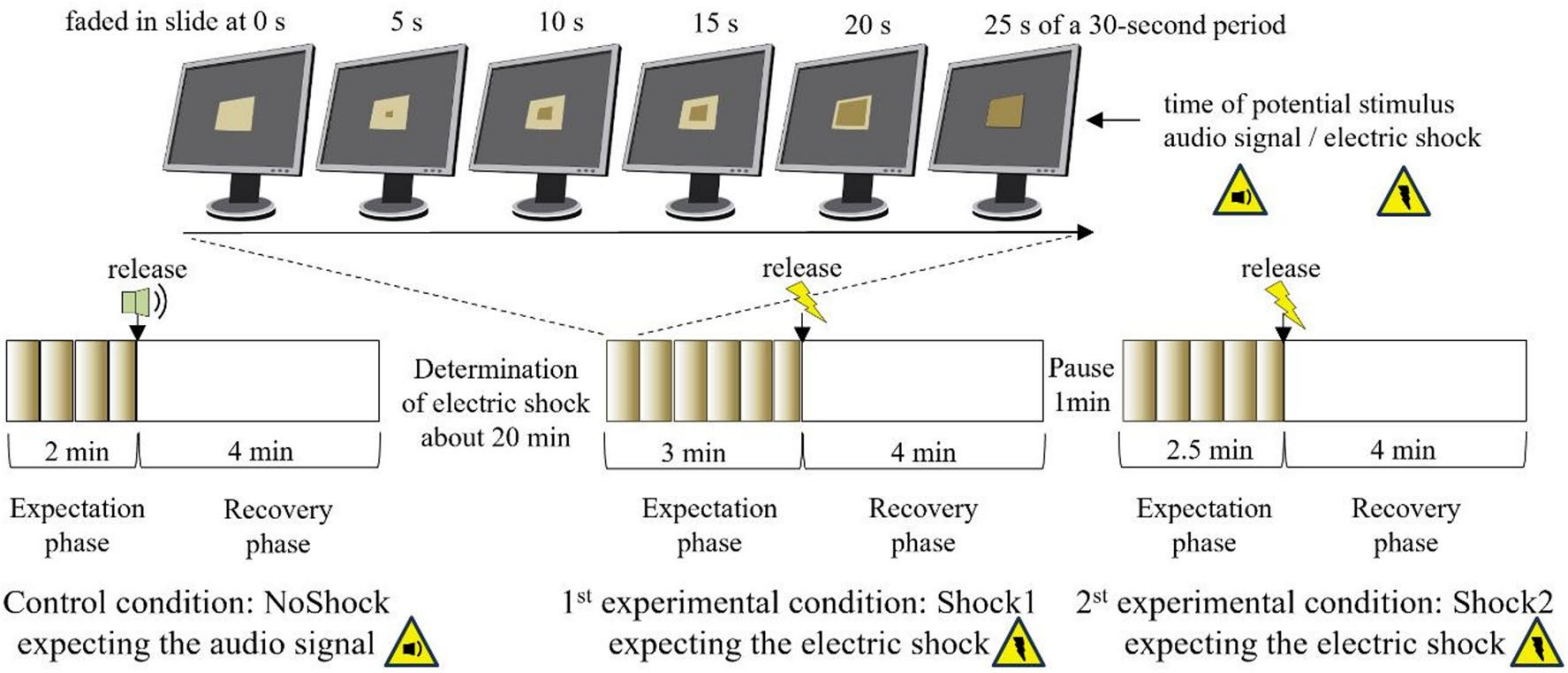
Measurement procedure of the three sequential experimental conditions slightly modified from Wagenblast et al.^49^ (Creative Commons Attribution 4.0 License). Conditions: NoShock with expectation of an audio signal, Shock1 with expectation of the first electric shock, and Shock2 with expectation of the second electric shock, together with the determination of the individual electric shock level and the slide show presenting the recurrently filling rectangles on the monitor during each period. Note, participants were unaware of the exact point in time when the stimulus (audio signal or electric shock) would be applied.

The duration of the expectation phases differed to prevent participants from anticipating the release of the electric shock (NoShock 2 min, Shock1 3 min, Shock2 2.5 min). The expectation phases were divided into 30s periods, with the first period lasting 35s and the final period ending after 25s with the release of the stimulus. Within periods, a rectangle was displayed on the screen that continued to fill every 5s until it was completely filled after 25s. Except in the first 35s period, where the rectangle started to fill after 10s, and in the final 25s period, where the stimulus was triggered after 25s (Fig. 1). This allowed for a gradual increase in stress anticipation every 5s and an event-related calculation of the skin resistance response at the times when the stimulus can be triggered.

### Instructions to the participants about the application of the stimuli

Participants were informed that the stimulus could be triggered by a computer program within the next four minutes, but only if the rectangle was completely filled. It was further explained that for Shock1 and Shock2, the intensity of the electric shock is randomly determined by the computer program and can range between the sensation threshold and clearly above the pain threshold to ensure that participants assume the possibility of pain, although the participants did not know that the intensity of the applied electric shocks was always determined by Eq. (1).

### Electric shocks

For the electric shocks, rectangle pulses with a duration of 2ms were applied with an electro-stimulator (DS7A, Digitimer Ltd., UK) and two Ag/AgCl cup electrodes filled with conductive paste (Ten20, Weaver and Company, USA). They were attached with adhesive tape (Fixomull stretch, BSN medical, Germany) to the distal phalanx of the index and middle finger of the non-dominant hand.

To keep the perceived electric shock intensity constant for Shock1 and Shock2 and to ensure that the application was perceived as painful but not harmful, the Eq. (1):

1 $$Electric{\text{ }}shock = pain{\text{ }}threshold + {\text{ }}0.{\text{25 }}*{\text{ }}\left( {pain{\text{ }}threshold{-}sensation{\text{ }}threshold} \right)$$

was adopted from Luijcks et al.^51^. Sensation and pain thresholds were determined twice in a two-step process described in Wagenblast et al.^49^, averaged, and used in the equation.

### Heart rhythm and skin resistance

*Heart rhythm* After preparing the skin with abrasive paste (Nuprep, Weaver and Company, USA) to improve signaling, pre-gelled Ag/AgCl surface electrodes (H93SG Kendall, Covidien, Ireland) were connected to the PS11 device (THUMEDI GmbH&Co.KG, Germany) and attached to the body starting with a neutral electrode at the 7th cervical vertebra.

Two electrodes were attached about 2 cm caudal and about 1 cm left-lateral from the cranial end of the sternum and to the intercostal space between the 4th and the 5th rib over the anterior axillary line to measure the electrical activity of the heart using bipolar ECG. The signal was recorded continuously by the measuring device at 1000 Hz and processed in real-time to calculate heart rate [bpm] and the beat-to-beat RR intervals [ms]. Then, the last two minutes of the expectation phases were extracted, as this corresponds to the duration of the shortest expectation phase (NoShock). The beat-to-beat RR intervals were checked for artefacts discussed by Marchant-Forde, et al.^52^. Erroneous intervals were excluded and replaced by quadric interpolation (polynomial 2nd order) or deviation using MATLAB R2017a (MathWorks, USA) (Supplementary Figure S1, S2). For the corrected data of the expectation phases two exclusion criteria were defined: more than 10% of the beat-to-beat RR intervals interpolated, and a temporal discrepancy between cumulative time of the RR intervals and 2-minutes greater than twice its mean RR interval time. Subsequently, the corrected intervals were processed with the HRV software Kubios (Standard 3.3.1, University of Kuopio, Finland) to calculate the HRV parameters: RMSSD [ms] from the time-domain and HF (ms^2^), and LF (ms^2^) from the frequency-domain using fast Fourier transform. While RMSSD and HF reflect parasympathetic or vagal activity, LF has been linked to the sympathetic impact on HRV^53^.

*Skin resistance* Two electrodes were placed on the non-dominant hand on the palmar sides of the middle phalanges of the little finger and the ring finger. A constant current of 11.5 ± 0.063 µA was applied by the measurement device (measurement range 50−1000k Ohm with an accuracy of ± 0.11%, resolution 0.26 k Ohm, ) to continuously record the voltage. Subsequently, SR was calculated and converted to the unit µS using the Eq. (2)

2 $$skin{\text{ }}resistance = voltage/constant{\text{ }}current$$

**Fig. 2:**
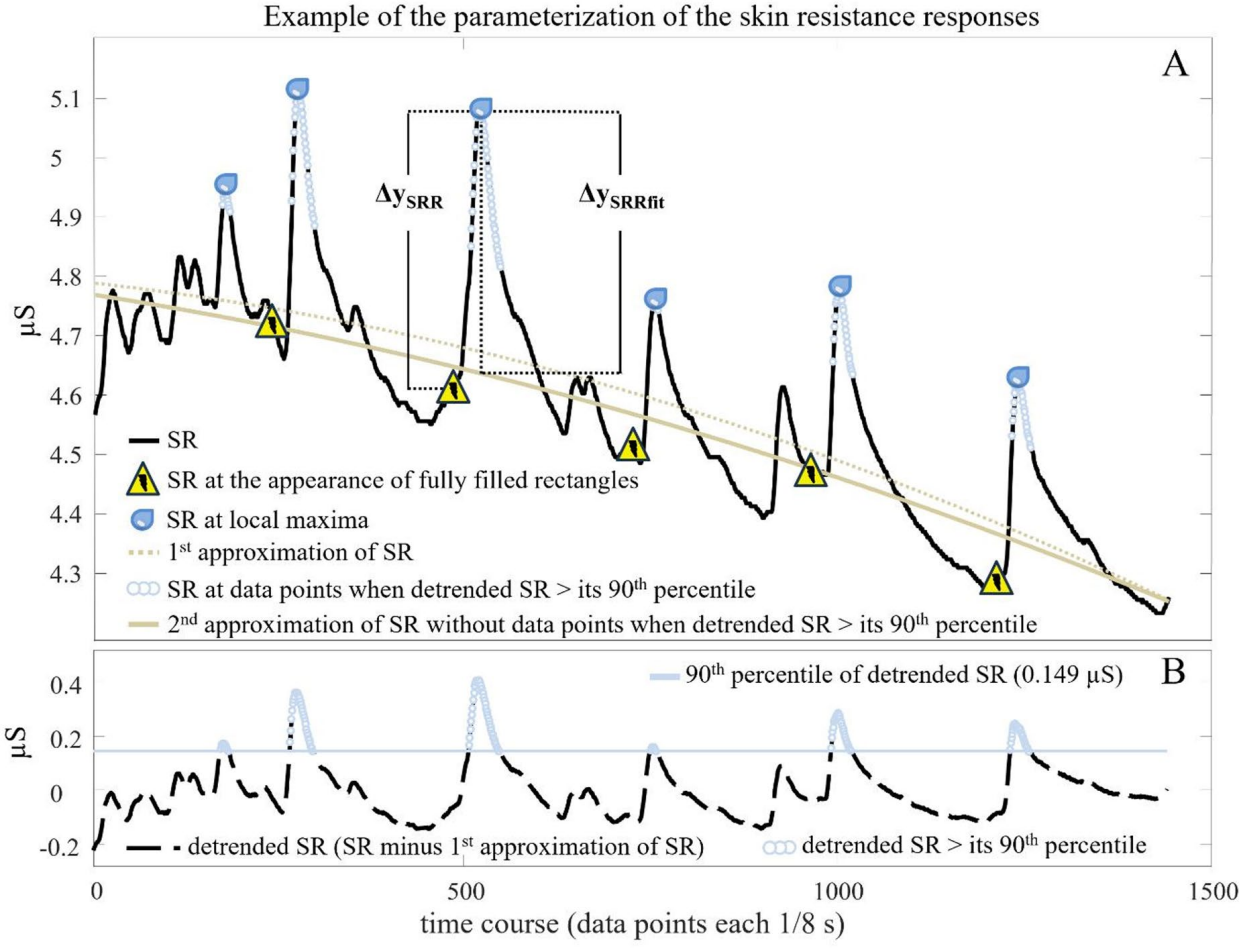
Example of the parameterization of the skin resistance responses during the expectation phase of the 1st electric shock. (**A**) Example of skin resistance (SR) values over time, appearance of the fully filled rectangles indicating the possibility of a stimulus, the local SR-maxima within 10s after the appearance of the stimuli, the 1st approximation (2nd order) of the SR, the SR at time points when the detrended SR is above its 90th percentile, the 2nd approximation (2nd order) of SR without data points above the 90th percentile of the detrended SR, the visualization of the parameterization of SRR and SRRfit as Δy_SRR_ and Δy_SRRfit;_ (**B**) Example of the time course of detrended SR (SR subtracted by the 1st approximation (2nd order) of the SR), with the 90th percentile of detrended SR and data points above the 90th percentile.

The parameter of the SR level (SRL) was defined as the median SR during expectation phases. Two SR response parameters (SRR and SRRfit) were determined by the average of all SR responses elicited by the fully filled rectangles that appeared on the screen during an expectation phase. SRR was calculated as the Δy_SRR_ between the SR value at the appearance of the fully filled rectangle that indicates the possibility of a stimulus and the maximum SR within the subsequent 10s. SRRfit was computed by a more explorative approach, which considered the downward drift of SR over time^54^. Therefore, SR during expectation phases was approximated (2nd order). The approximated values were subtracted from SR values for each time point to detrend the signal. All SR values at time points when the detrended SR is above its 90th percentile during an expectation phase were identified and excluded before a 2nd approximation (2nd order) was done. The 2nd approximation was calculated, because in many cases the 1st approximation was clearly higher than SR at the time the rectangle was fully filled. Δy_SRRfit_ was defined as difference between the 2nd approximation and SR at the time point of the maximum SR within 10s after the appearance of the fully filled rectangle (Fig. 2). Calculations of SR and SR parameters were done after recordings using MATLAB (R2017a, MathWorks, USA). All SR parameters are associated with sympathetic nervous system activation^25^.

### Neuroticism

The Big Five Inventory-SEOP^55^ was used to split the participant sample into two groups of lower (LowNeuro) and higher (HighNeuro) neuroticism. The personality trait neuroticism was assessed on the basis of the mean value of the agreement to three statements (Supplementary Table S1) rated on Likert scales from 1 to 7: 1 = “not true at all” and 7 = “totally true”. Participants were allocated to LowNeuro if their mean value were equal to or lower than the median of the sample, otherwise they were allocated to HighNeuro.

### Statistical analyses

Since studies suggest that responses of heart rhythm and EDA can be influenced by gender and age^56–59^, neuroticism groups were compared to identify different distributions of gender and age using Fisher’s exact test (gender) and Wilcoxon rank-sum tests (age). In case of statistically heterogeneous distributions, the responses (NoShock minus Shock1) in heart rhythm and SR parameters were examined for gender differences (Wilcoxon rank sum tests) and correlations with age (Spearman’s ρ).

In order to statistically identify responses during the expectation phases of the electric shocks and to determine whether these responses are moderated by the personality trait neuroticism, two-factor mixed analyses of variance (ANOVA) were applied to the dependent variables of the heart rhythm (mHR, RMSSD, HF, LF) and SR (SRL, SRR, SRRfit). The within-subject factor was condition (NoShock, Shock1, Shock2) and the between-subject factor, neuroticism (LowNeuro, HighNeuro).

Prior to the application of the ANOVAs all dependent variables were checked visually for normal distribution, sphericity using Mauchly’s sphericity test, and the assumption of homogeneity of variances using Levene’s test. Abnormally distributed dependent variables HF, LF, SRR, and SRRfit were logarithmized for further analyses. The results of SRL, SRR, and SRRfit had to be adjusted by Greenhouse-Geisser correction due to violations of sphericity.

The partial eta squared (η_p_^2^) was calculated for main and interaction effects of the two-factor mixed ANOVAs and interpreted as a small effect for values between 0.010 and 0.059, as a medium effect for 0.060–0.139, and as a large effect equal or above 0.140^60^. Post-hoc analyses were performed using Tukey’s Honestly Significant Difference tests.

The level of significance was set at α = 0.05 and adjusted to α = 0.007 for the ANOVAs using Bonferroni’s correction for multiple testing. The statistical analyses were carried out with SPSS (26, IBM, USA) and JMP (13, SAS Inc., USA).

## Results

Two of the 53 recruited participants dropped out due to discomfort and two for breaching the protocol (closed eyes, inability to follow instructions). Eight additional participants were excluded from the analyses of RMSSD, HF, and LF due to quality criteria of the HRV data correction. Since the 10% limit of erroneous data is an arbitrary threshold, the statistical analysis was also performed with a stricter 5% threshold. The 5% threshold did not change the statistical results, but resulted in the exclusion of two more participants. This reinforced the decision to keep the threshold at 10%, as it contributes to higher statistical power. Hence, exclusions differed between measurements. Therefore, it was decided to present the characteristics of the participants in Table 1 and the statistical comparisons of the gender and age distributions in Table 2 separately for mHR and the SR parameters versus the HRV parameters.

**Table 1:** Characteristics of participants considering lower and higher neuroticism groups and different samples used to analyze the parameters of heart rhythm and skin resistance.

Sample	Group	Gender (*n*) (women/men)	Neuroticism (mean value)	Age (years)	BMI (kg/m^2^)
Complete sample	No differentiation	49 (28/21)	3.4 (1.3)	34.7 (14.4)	23.0 (3.1)
Sample for analyses of mHR, SRL, SRR, SRRfit	LowNeuro	27 (11/16)	2.5 (0.8)	37.3 (13.1)	23.6 (3.2)
	HighNeuro	22 (17/5)	4.5 (0.8)	31.5 (15.5)	22.3 (3.0)
Sample for analyses of RMSSD, HF, LF	LowNeuro	23 (9/14)	2.5 (0.8)	34.3 (11.8)	23.5 (3.3)
	HighNeuro	18 (15/3)	4.5 (0.8)	31.1 (15.6)	22.1 (2.7)

Mean (standard deviation) for neuroticism, age, and body mass index (BMI) in groups of lower (LowNeuro) and higher neuroticism (HighNeuro); mHR, mean heart rate; RMSSD, root mean square of successive differences of the time period between normal heartbeats; low (LF) and high (HF) frequency domains of the heart rate variability; SRL, skin resistance level; SSR, skin resistance response; SRRfit, detrended skin resistance response.

In both samples, the neuroticism groups showed significant differences in their gender distributions, whereas age distributions were only different between neuroticism groups in the sample for the analyses of mHR and the SR parameters (Table 2). However, there were no statistical differences between women and men in the physiological responses to the 1. expectation phase. Only responses of the SRR and SRRfit parameters correlated significantly with age (Table 3).

**Table 2:** Comparison of gender and age distributions between groups of lower and higher neuroticism considering the different samples used to analyze the parameters of heart rhythm and skin resistance.

Sample	Variable	Group comparison	Z-value	*p*-value
Sample for analyses of mHR, SRL, SRR, SRRfit	Gender ^a^	LowNeuro vs. HighNeuro	–	0.019*
	Age ^b^	LowNeuro vs. HighNeuro	2.2	0.029*
Sample for analyses of RMSSD, HF, LF	Gender ^a^	LowNeuro vs. HighNeuro	–	0.010*
	Age ^b^	LowNeuro vs. HighNeuro	1.9	0.063

Results of statistical tests used to compare the distributions of gender: Fisher’s exact test ^a^, and age: Wilcoxon rank-sum test ^b^, between groups of lower (LowNeuro) and higher neuroticism (HighNeuro); mHR, mean heart rate; RMSSD, root mean square of successive differences of the time period between normal heartbeats; low (LF) and high (HF) frequency domains of heart rate variability; SRL, skin resistance level; SSR, skin resistance response; SRRfit, detrended skin resistance response; *significant *p*-value < 0.05.

**Table 3:** Statistical tests of gender and age effects on physiological responses to the expectation of the 1st shock in relation to parameters of heart rhythm and skin resistance.

Parameter (response to 1st electric shock)	Gender ^a^		Age ^b^	
	Z-value	*p*-value	*r*	*p*-value
mHR	− 1.67	0.096	− 0.09	0.556
RMSSD	− 0.99	0.321	N/A	N/A
HF	− 1.81	0.070	N/A	N/A
LF	− 0.94	0.341	N/A	N/A
SRL	− 1.12	0.262	− 0.06	0.658
SRR	− 0.90	0.369	− 0.54	< 0.001*
SRRfit	− 0.56	0.579	− 0.53	< 0.001*

Results of Wilcoxon rank-sum tests ^a^ (gender) and Spearman’s correlations ^b^ (age), examining significant effects of gender or age on heart rhythm and skin resistance parameters in response to the first shock expectation; a negative z-value indicates a response towards higher strain in women; a negative r indicates a negative relationship between a response towards higher strain and age; mHR, mean heart rate; RMSSD, root mean square of successive differences of the time period between normal heartbeats; low (LF) and high (HF) frequency domains of the heart rate variability; SRL, skin resistance level; SSR, skin resistance response; SRRfit, detrended skin resistance response; *significant p-value < 0.05; N/A not applicable due to non-significant differences in age between groups of lower and higher neuroticism.

The two-factor mixed ANOVAs identified significant main effects of condition with medium to large effect sizes for the parameters LF, SRL, SRR and SRRfit. Additionally, mHR showed a significant interaction effect between condition and neuroticism of medium effect size. There were no significant main effects of neuroticism (Table 4, Supplementary Table S2).

**Table 4:** Results of two-factor mixed ANOVAs applied to parameters of heart rhythm and skin resistance.

Parameter	Effect	df, df residuals	F-value	*p*-value	Partial eta-squared
mHR	Condition	2.0, 94.0	4.0	0.022	0.078
	Neuroticism	1.0, 47.0	0.7	0.414	0.014
	Condition*neuroticism	2.0, 94.0	5.7	0.005*	0.108
RMSSD	Condition	2.0, 78.0	4.0	0.022	0.093
	Neuroticism	1.0, 39.0	1.5	0.223	0.038
	Condition*neuroticism	2.0, 78.0	1.7	0.191	0.042
logHF	Condition	2.0, 78.0	0.5	0.595	0.013
	Neuroticism	1.0, 39.0	2.9	0.095	0.070
	Condition*neuroticism	2.0, 78.0	2.9	0.059	0.070
logLF	Condition	2.0, 78.0	12.6	< 0.001*	0.244
	Neuroticism	1.0, 39.0	0.2	0.693	0.004
	Condition*neuroticism	2.0, 78.0	0.9	0.410	0.023
SRL	Condition ^a^	1.5, 69.1	53.4	< 0.001*	0.532
	Neuroticism	1.0, 47.0	0.5	0.484	0.010
	Condition*neuroticism ^a^	1.5, 69.1	3.5	0.050	0.069
logSRR	Condition ^a^	1.7, 77.6	37.2	< 0.001*	0.442
	Neuroticism	1.0, 47.0	0.1	0.761	0.002
	Condition*neuroticism ^a^	1.7, 77.6	0.1	0.838	0.003
logSRRfit	Condition ^a^	1.7, 79.6	38.5	< 0.001*	0.450
	Neuroticism	1.0, 47.0	< 0.1	0.825	0.001
	Condition*neuroticism ^a^	1.7, 79.6	< 0.1	0.976	< 0.001

**Fig. 3:**
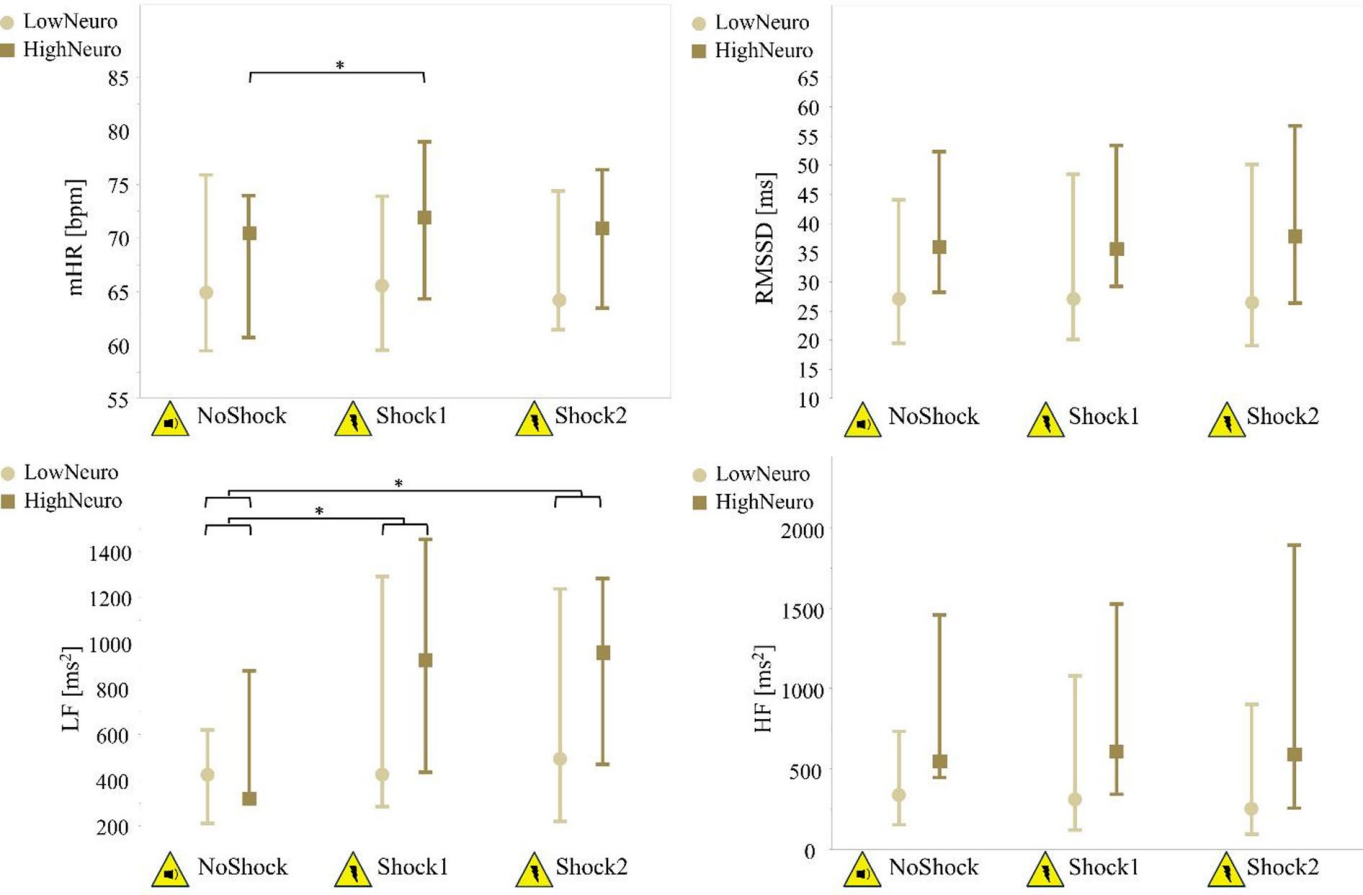
Results of heart rhythm parameters during three conditions. Medians and interquartile ranges of the parameters mHR (mean heart rate), RMSSD (root mean square of successive differences of the time period between normal heartbeats), LF, and HF (low and high frequency domains of the heart rate variability) during the following conditions: expecting audio signal (NoShock), expecting first electric shock (Shock1), and expecting second electric shock (Shock2) (Supplementary Table S2); brighter circles represent the group with lower neuroticism (LowNeuro), darker squares represent the group with higher neuroticism (HighNeuro); *significant *p*-value < 0.05 of the post hoc comparisons.

**Fig. 4:**
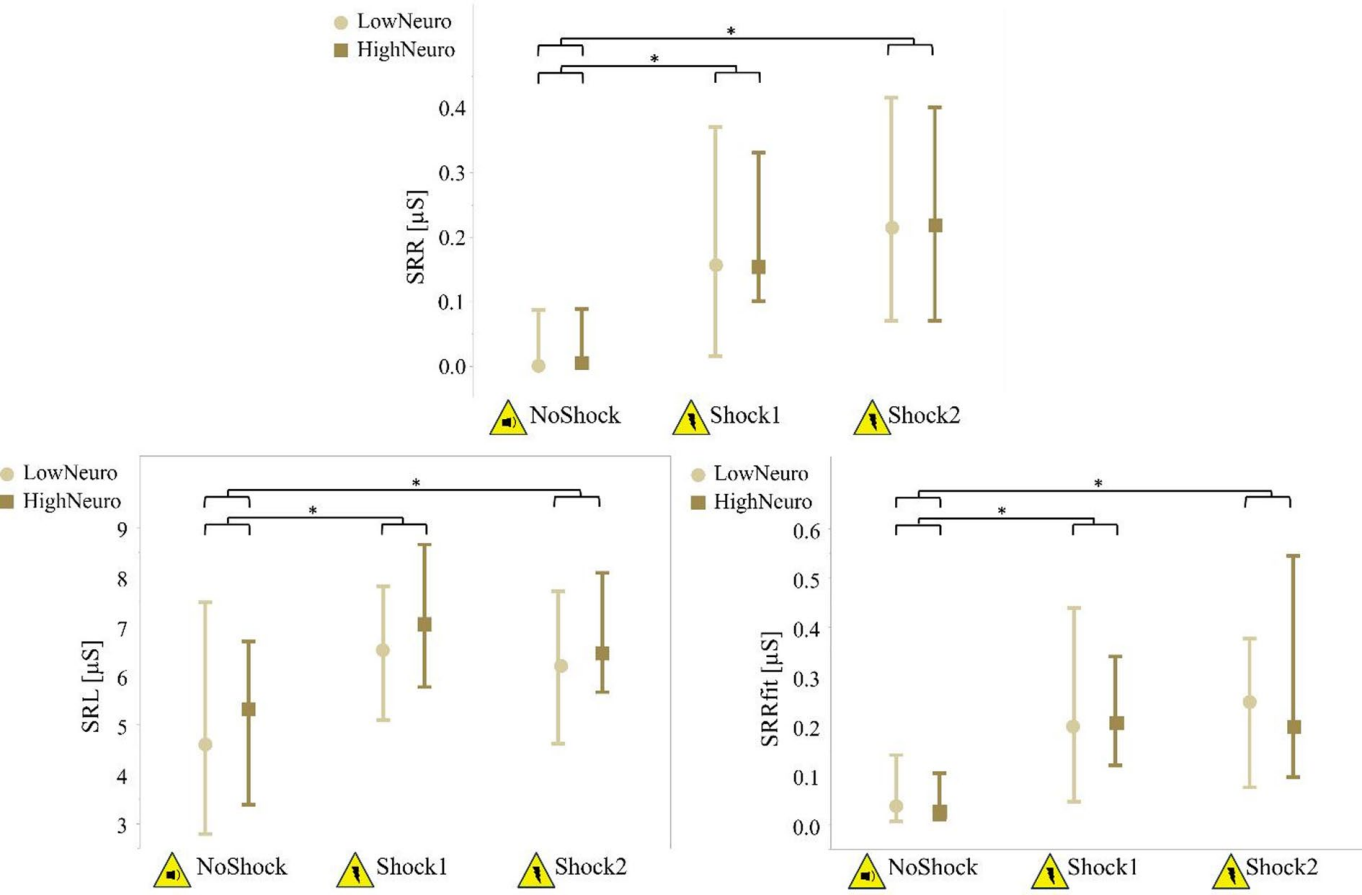
Results of skin resistance parameters during three conditions. Medians and interquartile ranges of the parameters SRL (skin resistance level), SRR (skin resistance response), and SRRfit (detrended skin resistance response) during conditions: expecting an audio signal (NoShock), expecting first electric shock (Shock1), and expecting second electric shock (Shock2) (Supplementary Table S2); brighter circles represent the group with lower neuroticism (LowNeuro), darker squares represent the group with higher neuroticism (HighNeuro); *significant p-value < 0.05 of the post hoc comparisons.

Parameters of heart rhythm and skin resistance (mHR, mean heart rate, RMSSD, root mean square of successive differences of the time period between normal heartbeats, logLF and high logHF, logarithmized low and high frequency domains of the heart rate variability, SRL, skin resistance level, logSSR, skin resistance response, logSRRfit, detrended skin resistance response) derived from the expectation phases as the dependent variables for the two-factor mixed ANOVAs with the within-factor condition (NoShock with audio signal, Shock1 with first electric shock, and Shock2 with second electric shock) and the between-factor neuroticism (groups of lower and higher neuroticism defined by a median split); ^a^ corrected for violation of sphericity via Greenhouse-Geisser adjustment; *significant *p*-value < 0.007;

The post-hoc comparisons of the main effect of condition revealed a significantly higher LF, SRL, SRR, and SRRfit during Shock1 and Shock2 compared to NoShock (*p*-values < 0.001), while Shock1 and Shock2 were not significantly different. The post-hoc comparisons of the interaction effect between condition and neuroticism on mHR revealed that only HighNeuro, responded to Shock1 with a significant increase from NoShock to Shock1 (*p* = 0.001), but showed no significant changes in Shock2 compared to NoShock and Shock1 (Figs. 3 and 4, Supplementary Table S2).

A complementary analysis of mental tension ratings was published by Wagenblast et al.^49^. Mental tension was rated on a numeric rating scale (from 0 to 10 with 0 = no mental tension and 10 = highest imaginable mental tension) before each expectation phase. Compared to NoShock (mean ± SD 1.5 ± 1.3), Shock1 (3.6 ± 2.0) and Shock2 (2.7 ± 1.6) was rated higher. Also, Shock1 was rated higher as Shock2.

## Discussion

The primary aim of the study was to investigate reactivity of heart rhythm and skin resistance parameters to recurring anticipatory stress and secondarily whether reactivity depends on the personality trait neuroticism. Knowledge on which parameter are more or less robust against changes over time effects or influenced by the personal trait neuroticism may help to develop the best approach for supplementing the risk assessment of mental stress at the workplace by objective psychophysiological parameters. SR and HRV parameters associated with the sympathetic nervous system responded independently to the expectation of the 1st electric shock, without an adaption in responsiveness during the expectation of the 2nd electric shock, whereas HRV parameters associated with the parasympathetic nervous system did not respond. A HR response was detected only to the 1st electric shock in the group with higher neuroticism.

### Recurrent anticipation of an electric shock

RMSSD and HF consistently showed no responses to the expectation of the 1st or 2nd electric shock. In contrast, some studies observed lower RMSSD or higher HF during the anticipation of or presence of a stressor. Pulopulos et al.^61^ and Wearne et al.^62^, for example, found a lower HRV during the Trier Social Stress Test compared to a baseline phase. Although the Trier Social Stress Test distinguishes between the anticipation of a stressor (preparing to deliver a speech) and the presence of a stressor (delivering a speech), the results of the anticipation phase cannot be compared precisely with those of the present experiment. During the present experiment, participants sat still with no cognitive or executive functions, except for attention, which was intended to be kept constant across conditions by presenting rectangles on the screen. This means that it cannot be clearly determined whether the decreased HRV during the anticipation of delivering a presentation is attributed to a negative affect or cognitive workload, such as attentional demands^63^. Alternatively, there is the possibility that the threat of social evaluation, representing a psychosocial stressor, contributes to a higher parasympathetic response, in contrast to the threat of a painful stimuli^64^, given that in the present experiment the perceived mental tension directly before the 1st expectation phase of the electric shock was rated as low to moderate. The SR parameters presented responses to the 1st without reductions to the 2nd expectation of the electric shock, suggesting no adaptation effects. This is in line with the findings from Holmes and Houston^65^, who found increased skin resistance in anticipation of a painful electric shock. Since the experiment was designed to control for temperature, executive functions, and attention, it could be suggested that during both exposures the SR response represented increased sympathetic activation in the context of negative valence that was accompanied by a behavioral response-focused “fight-or-flight” strategy^66^. This interpretation is supported by the increased mental tension before both electric shock conditions^49^ and studies that use unpredictable/predictable electric shock paradigms to trigger negative emotions^67,68^. The absence of strong, statistically significant adaptation effects might be attributed to the unknown intensity and moment of application of the electric shock, which did not allow for an exact threat prediction in terms of the significance of pain and the resultant impact on homeostasis. In line with these results, Ring and Kaernbach^69^ demonstrated that SC responses rise with a higher probability of receiving an electric shock. Additionally, LF responded in a comparable way, although the influence of the cardiac rhythm and baroreceptors on the interaction of sympathetic and parasympathetic reactivity do not appear to be related to EDA responses^70^. Predominantly based on the relationship between heart rate and parasympathetic activity, Billman^71^ emphasized that there are substantial concerns regarding a linear relation between LF and sympathetic reactivity. Moreover, from a physical point of view, LF at 0.04 Hz require periods of at least 25s to reliably extract spectral information. However, the quality of measurement can be compromised if the recorded time is too short and limited information of a few periods can be used only for an approximation^72^.

From a practical point of view, these results imply that the applied methods and parameters associated predominantly with the response of the sympathetic nervous system are sensitive to the same recurrent anticipatory stress independently from the personality trait neuroticism. Therefore, these methods seem to be more suitable for the identification of mental stressors, although their applicability in the field requires further validation. Compared to LF, the SR parameters offer the advantage of capturing the stressor more precisely in terms of time because the temporal resolution is higher. Precisely determining the frequency with which a stressor occurs in the workplace could help to more accurately assess mental stress and associated potential health risks.

### Interactions between recurrent anticipation of an electric shock and neuroticism

None of the parameters showed interaction effects between a recurrent anticipation of the electric shock and neuroticism, except the mean heart rate. The group with higher neuroticism responded with an increase in mean heart rate to the expectation of the 1st electric shock but not during the expectation of the 2nd electric shock, whereas the group with lower neuroticism did not respond to either. Hypothetically, the response could be explained by the baroreflex, which might have been activated by an initial drop in arterial blood pressure at the beginning of 1st electric shock expectation. Consequently, the vasoconstriction increased by a disinhibition of the sympathetic neural outflow via a decrease of the afferent input to the central autonomic nuclei and resulted in an increased HR. According to the Defense Cascade model, it could be assumed that during a post-encounter phase, before the fight-or-flight response, the emotional intensity increases and so does, for example, the SR, but the HR would decrease up until a certain threshold of emotional intensity is reached and only then increase^73^. The threshold may have been reached only by the group with higher neuroticism, even if the response of the SR showed no difference between groups. The subsequent absence of effects regarding the expectation of the 2nd electric shock in the group with higher neuroticism might be related to initialization mechanisms of vagal control. This assumption is based on a trend of increased parasympathetic activity indicated by the RMSSD during the expectation of the 2nd electric shock, when compared to the expectation of the audio signal. In contrast, Hughes et al.^74^ found a lower HR response due to higher neuroticism and no adaption effect during two consecutive exposures to a mental arithmetic task on the computer. However, Jonassaint et al.^75^, who investigated cardiovascular reactivity during an arithmetic mental task and emotional stress (anger recall) with regard to neuroticism, proposed that blunted responsiveness due to higher neuroticism may be attributed only to non-emotional stress, for example.

### Strength and limitations

Beginning with the strengths of the study, it should be noted that the elaborated methodological issues have been rarely addressed in the context of a work-related risk assessment of mental stress. In addition, the relatively large sample size of *n* = 53 increases the significance of the results, which appear to be stringent in terms of content and methodology and thus provide a basis for further methodological studies in this area.

Even though parameter responses were not statistically significantly influenced by gender, limitations might have resulted from the group splitting based on the median of neuroticism values. A remarkable difference is the gender ratio, with only three males out of 18 participants in the group with higher neuroticism for the analysis of the HRV parameters, which is in line with the literature regarding a more prominent personality trait of neuroticism in women^76^. Research findings suggest that the autonomous stress response can depend on the sex-specific hormonal status^77^. Considering that the status of the menstrual cycle or the use of contraceptives of female participants was not surveyed, it cannot be fully ruled out that certain physiologic responses are biased. However, these sex effects should be canceled due to accidental sampling.

SRR and SRRfit were significantly negatively correlated with age, implying reduced sympathetic response in older adults and therefore potentially biasing the present results towards an attenuated response in the group with higher neuroticism, which represented a statistically significant lower age when SR parameters were analyzed. A reduced responsiveness due to age has been reported in literature and was attributed to peripheral (smaller number of active sweat glands) and central (reduction in gray matter) nervous system changes in older adults^78–80^. However, in this regard it is questionable if the significant, but minor age differences between the present neuroticism groups might have substantial implications for the present results.

Work conditions, particularly, physical stress or temperature can influence psychophysiological parameters under real-world conditions. Therefore, a limitation of our study is that we tested the responsiveness of the parameters under standardized laboratory conditions without considering the influence multiple stressors.

## Conclusion

In summary, results of psychophysiological methods and parameters differed when the same anticipatory stress paradigm was applied twice to the same individuals with either low or high neuroticism. Parameters associated predominantly with the sympathetic nervous system showed similar responses to the 1st and 2nd electric shock exposure, whereas parasympathetic associated parameters didn’t respond at all. Contrary, the HR responses were influenced by personality. It is concluded that the application of the parameters associated with a sympathetic activity may allow a more accurate mapping of anticipatory stressors. Together with subjective assessment tools, this could contribute to a more comprehensive risk assessment of mental stress at work.

The here presented responding parameters should be further evaluated on their responsiveness to other mental stress scenarios, regarding their practicability, susceptibility to interference and long-term application (e.g. a complete work shift).

## Supplementary Information

Below is the link to the electronic supplementary material.

Supplementary Material 1

## Data Availability

The datasets generated during and/or analysed during the current study are available from the corresponding author Florestan Wagenblast on reasonable request.
