# Supplementary material for: Measures of the psychophysiological response to recurrent anticipatory stress - the influence of neuroticism on heart rhythm and skin resistance
Source: Sci Rep. 2025 Nov 21;15:41177. doi: 10.1038/s41598-025-28090-7 (PMC12638765; doi:10.1038/s41598-025-28090-7)
Supplement: Supplementary file 1 — Supplementary Material 1 [file 41598_2025_28090_MOESM1_ESM.docx]

## Supplementary Information

Supplementary Figure S1

Error type classification according to Marchant-Forde et al. (2004)


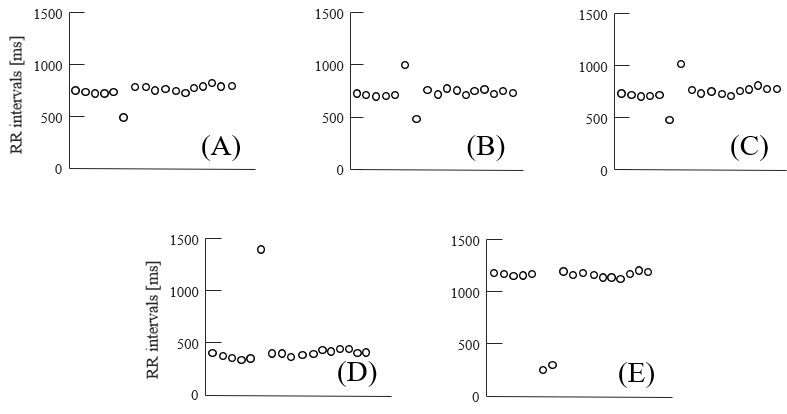


Time course of RR-intervals illustrating error types 1 to 5. (A) Type 1: single RR interval discrepancy, (B) Type 2: long RR interval followed by a short RR interval with similar deviations, (C) Type 3: short RR interval followed by a long RR interval with similar deviations, (D) Type 4: double to triple length of single RR interval, (E) Type 5: two or more short RR intervals.

Marchant-Forde, R. M., Marlin, D. J. & Marchant-Forde, J. N. Validation of a cardiac monitor for measuring heart rate variability in adult female pigs: accuracy, artefacts and editing. *Physiol Behav* **80**, 449-458 (2004). <https://doi.org:10.1016/j.physbeh.2003.09.007>

Supplementary Figure S2

Visual example of identification of the error type with the corresponding data correction procedure


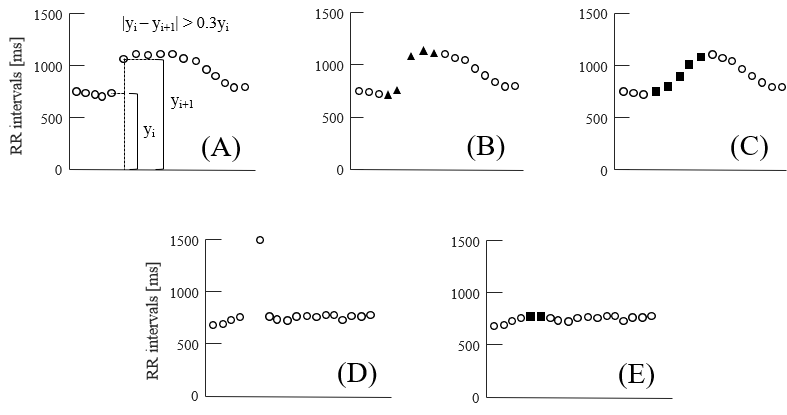


For type 1, 2, 3, and 5 errors: (A) if the absolute difference (|y_i_-y_i+1_|) between two successive RR-intervals is > 30% of the first RR interval, (B) then the erroneous RR interval plus the two previous and following RR intervals (triangles) are deleted, (C) and replaced (rectangles) using quadratic interpolation (polynomial 2^nd^ order). For type 4 errors: (D) if an RR interval is two or three times the mean of the previous and following RR intervals, (E) then the erroneous RR interval is split into two or three RR intervals of equal size (rectangles).

Supplementary Table S1

Three statements of the German Big Five Inventory-SEOP translated into English:

I am someone who …

not true totally

at all true

| worries a lot. | 1 2 3 4 5 6 7 |
| --- | --- |
| gets nervous easily. | 1 2 3 4 5 6 7 |
| is relaxed, and copes well with stress.* | 1 2 3 4 5 6 7 |

* statement formulated against the direction of the construct neuroticism

Supplementary Table S2

Median and interquartile range (IQR) of heart rhythm and skin conductance parameters.

| **Parameter** | **Group** | **Condition** | | |
| --- | --- | --- | --- | --- |
|  |  | **NoShock** | **Shock1** | **Shock2** |
| mHR [bpm] | LowNeuro | 65.1 (16.4) | 65.7 (14.6) | 64.4 (13.0) |
|  | HighNeuro | 70.6 (13.2) | 72.1 (14.3) | 71.1 (12.9) |
| RMSSD [ms] | LowNeuro | 27.2 (25.8) | 29.0 (30.2) | 29.2 (32.5) |
|  | HighNeuro | 35.9 (24.6) | 35.8 (25.8) | 36.9 (31.8) |
| HF [ms^2^] | LowNeuro | 370 (612) | 334 (1061) | 265 (899) |
|  | HighNeuro | 613 (1152) | 661 (1302) | 581 (1675) |
| LF [ms^2^] | LowNeuro | 436 (373) | 485 (987) | 515 (1134) |
|  | HighNeuro | 337 (610) | 998 (903) | 964 (943) |
| SRL [µS] | LowNeuro | 4.66 (4.69) | 6.58 (2.71) | 6.25 (3.09) |
|  | HighNeuro | 5.37 (3.31) | 7.10 (2.89) | 6.51 (2.42) |
| SRR [µS] | LowNeuro | 0.005 (0.087) | 0.161 (0.356) | 0.219 (0.345) |
|  | HighNeuro | 0.009 (0.089) | 0.158 (0.231) | 0.223 (0.331) |
| SRRfit [µS] | LowNeuro | 0.032 (0.134) | 0.194 (0.393) | 0.243 (0.301) |
|  | HighNeuro | 0.020 (0.093) | 0.200 (0.220) | 0.193 (0.449) |

Median and interquartile range (IQR) of heart rhythm (mHR: mean heart rate; RMSSD: root mean square of successive differences of the time period between normal heartbeats; LF: low frequency domains of the heart rate variability; HF: frequency domains of the heart rate variability), and skin conductance parameters (SRL: skin resistance level; SSR: skin resistance response; SRRfit: detrended skin resistance response) for groups with lower (LowNeuro) and the higher neuroticism (HighNeuro)
